# Protection against the allergic airway inflammation depends on the modulation of spleen dendritic cell function and induction of regulatory T cells in mice

**DOI:** 10.1186/1479-0556-8-2

**Published:** 2010-03-24

**Authors:** Yaoli Wang, Chunxue Bai, Guansong Wang, Diane Wang, Xiaoming Cheng, Jian Huang, Dongpo Jiang, Guisheng Qian, Xiangdong Wang

**Affiliations:** 1 Institute of Respiratory Diseases, Xinqiao's Hospital, Third Military Medical University, Chongqing, China; 2 Department of Pulmonary Medicine, Zhongshan Hospital, Fudan University, Shanghai, China; 3 Intensive Care Unit, Daping's Hospital, Third Military Medical University, Chongqing, China

## Abstract

**Background:**

Allergen-induced imbalance of specific T regulatory (Treg) cells and T helper 2 cells plays a decisive role in the development of immune response against allergens.

**Objective:**

To evaluate effects and potential mechanisms of DNA vaccine containing ovalbumin (OVA) and Fc fusion on allergic airway inflammation.

**Methods:**

Bronchoalveolar lavage (BAL) levels of inflammatory mediators and leukocyte infiltration, expression of CD_11c_^+^CD_80_^+ ^and CD_11c_^+^CD_86_^+ ^co-stimulatory molecules in spleen dendritic cells (DCs), circulating CD_4_^+ ^and CD_8_^+ ^T cells, Foxp3^+ ^in spleen CD_4_^+ ^T cells and spleen CD_4_^+ ^T cells were measured in OVA-sensitized and challenged animals pretreated with pcDNA, OVA-pcDNA, Fc-pcDNA, and OVA-Fc-pcDNA.

**Results:**

OVA-Sensitized and challenged mice developed airway inflammation and Th2 responses, and decreased the proliferation of peripheral CD_4_^+^and CD_8_^+ ^T cells and the number of spleen Foxp_3_^+ ^Treg. Those changes with increased INF-γ production and reduced OVA-specific IgE production were protected by the pretreatment with OVA-Fc-pcDNA.

**Conclusion:**

DNA vaccine encoding both Fc and OVA showed more effective than DNA vaccine encoding Fc or OVA alone, through the balance of DCs and Treg.

## Introduction

Allergic asthma is a Th2 lymphocyte-associated inflammatory airway disease characterized by airway eosinophilia, goblet-cell hyperplasia, variable airway obstruction and hyper-responsiveness [1]. The balance between allergen-specific T regulatory (Treg) cells and T helper 2 cells appears to be decisive in the development of the immune response against allergens [2]. Allergen-specific immunotherapy (SIT) has been suggested as one of the few antigen-specific treatments for inflammatory diseases, with a long-term of efficacy [2]. SIT could reduce the development of asthma and bronchial responses in patients exposed to inhaled allergens. It is possible to target anti-inflammatory therapy to the various pathways of the disease, improving asthma control. However, the mechanisms by which allergen-DNA-targeted dentritic cells (DCs) plays anti-inflammatory roles remain unclear.

We found that allergen-DNA-targeted DCs reduced Th2 responses and the expression of C co-stimulatory molecules like D_11c_^+^CD_80_^+ ^and CD_11c_^+^CD_86_^+ ^in experimental asthma [3]. The present study furthermore investigated the potential mechanisms where Treg cells and spleen DCs may be involved in the therapeutic process of DNA vaccination coding with Fc and ovalbumin (OVA-Fc-DNA) in in allergic models. We determine the therapeutic role of immunization with OVA-Fc-DNA-targeted DCs and ascertain the roles of spleen DCs in the protection.

## Methods

### Animals

Male BALB/c mice, 6-10 weeks old at the onset of experiments, were purchased from Institute of Animal in Third Military Medical University (Chongqing, China). Animal care and experimental procedures were in accordance with the animal ethics regulations of the Home Office, UK.

### Construction of OVA-Fc-pcDNA_3.1 _immunization vector

**Figure 1:**
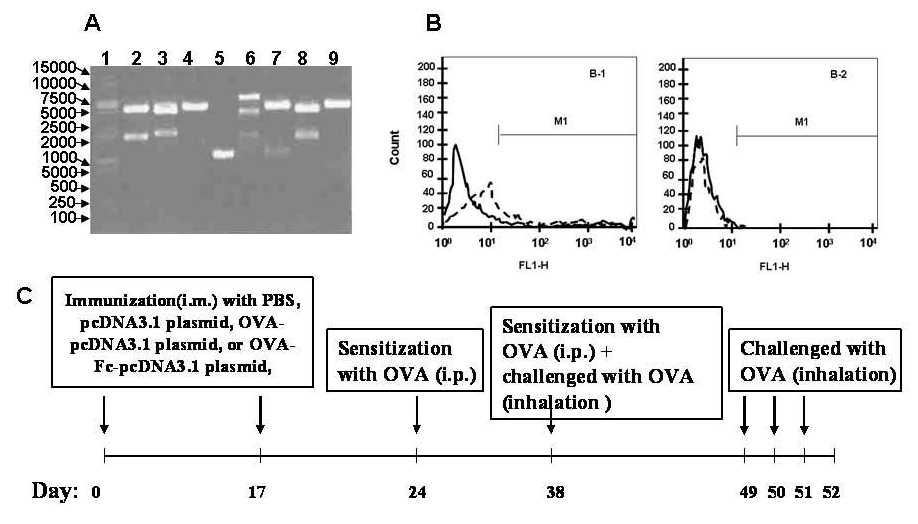
**Construction of recombinant plasmid OVA-Fc-pcDNA_3.1 _(A) includes 1: DL 2 000 + DL 15 000 marker; 2: OVA-pMIgV/EcoR I + Xba I; 3:OVA-Fc-pcDNA/B gl II; 4: OVA-Fc-pcDNA/EcoR I; 5: PCR product of OVA-pcDNA_3.1_; 6: λDNA/EcoR I + Hind III; 7: OVA-pMIgV/EcoR I +Bgl II; 8: OVA-Fc-pcDNA/EcoR I+Xba I; 9: OVA-pMIgV/Bgl II+Xba I**. The expression of OVA-Fc fusion protein was detected in OVA-Fc-pcDNA 3.1-transfected CHO cells (B-1) and pcDNA3. 1-transfected CHO cells (B-2) by flow Cytometry. Immunization scheme of DNA vaccination prevention of avalbulin (OVA)-induced allergic airway inflammation (C).

To construct the DNA vaccine containing OVA and Fc fusion gene targeting DCs, the murine OVAcDNA was amplified from OVA-pcDNA3.1 plasmid by polymerase chain reaction (PCR), spliced and then cloned into pMIgV containing murine IgG2a Fc cDNA. OVA-Fc-pcDNA3.1 plasmid was finally constructed after sub-cloning spliced OVA-Fc into pcDNA_3.1 _plasmid. OVA-Fc-pcDNA3.1 plasmids were then transfected into CHO cells with lipofectamine. The expression of OVA and Fc was determined by flow cytometry, Western blotting, and enzyme-linked immunosorbent assay (ELISA). DNA sequencing and restriction endonuclease digestion analysis indicated that the eukaryotic expression vector OVA-Fc-pcDNA_3.1 _had been constructed successfully. The expression of OVA and Fc expression could be detected in CHO cells by Western blotting, ELISA, and flow cytometry, as shown in Fig. 1A and 1B. The PCR products of OVA and Fc were selected as target DNA fragments and cloned into pcDNA_3.1 _(+) to construct the recombinant plasmids OVA-pcDNA_3.1 _and OVA-Fc-pcDNA_3.1 _respectively. The plasmids were propagated in Escherichia coli and large scale purification of all plasmids was conducted with the EndoFree Plasmid Giga Kit (Qiagen, Mississauga, Canada) according to the manufacturer's instructions.

### Immunization protocols

BALB/c mice (8-wk-old, male, 20~25 g) were maintained under standard conditions with free access to water and rodent laboratory food. Mice were handled according to experimental procedures involving mice [4]. Mice were divided randomly into the following groups, A: PBS or plasmid- vaccinated mice sensitized and challenged with PBS, B: PBS- vaccinated mice sensitized and challenged with OVA, C: pcDNA_3.1 _plasmid-vaccinated mice sensitized and challenged with OVA, D: OVA- pcDNA_3.1 _plasmid-vaccinated mice sensitized and challenged with OVA, and E: OVA-Fc-pcDNA_3.1 _plasmid-vaccinated mice sensitized and challenged with OVA (n = 8 per group). BALB/c mice were injected intramuscularly with 100 μg of PBS, pcDNA, OVA-pcDNA and OVA-Fc-DNA in a final volume of 100 μl 0.9% NaCl on day 0 and boosted intramuscularly with the same amount of plasmid DNA. The mice were then sensitized to OVA for the induction of allergic asthma in BALB/c mice, as described previously [5]. OVA (grade V, 50 mg) adsorbed to 2 mg aluminum potassium sulfate (alum) was administered intraperitoneally on days 24 and 38, followed by an inhalation of 1% OVA (grade II) diluted in PBS for 30 min on days 38, 49, 50, and 51, respectively. Control mice received the same processes with OVA and an inhalation of PBS for 30 min and experimental design was shown in Fig. 1C. Twenty four hours after the last challenge (day 52), mice were sacrificed, blood was taken, bronchoalveolar lavage (BAL) was performed, lungs were removed and fixed, and spleen DCs and CD_4_^+^T cells were isolated for in vitro culture.

### Determination of OVA- specific IgE levels in serum

Serum levels of OVA-specific IgE were determined by ELISA. Briefly, 96 microtiter plates were coated overnight with 100 μl of OVA (10 μg/ml in 0.1 mol carbonate buffer, pH 9.6) at 4°C. The antigen-coated plates were washed with PBST (0.5% Tween-20 in PBS) thrice. Mouse sera were added and the plates were incubated with peroxidase-conjugated anti-mouse IgE antibody (Southern Biotech, USA) at 4°C overnight, and then washed with PBST thrice before adding citric acid-phosphate buffer (pH 5.0) containing 0.5 mg/ml of O-phenylenediamine (Sigma, USA). Color was developed at 37°C and measured at 450 nm after the reaction was stopped with sulfuric acid at 2.5 mol/L.

### Bronchoalveolar lavage

The trachea were exposed, cannulated and gently instilled with 500 μl of cold PBS twice. The volume, total cell number and composition of BAL samples were recorded. Samples were centrifuged (× 500 g for 5 minutes at 4°C), resuspended and cytospined onto slides. Different cells from each sample were counted for 200 cells in duplicate on coded slides. BAL fluid was stored at -70°C and levels of the cytokines interleukin (IL)-4, IL-5 and interferon (INF)-γ were determined using specific ELISA according to the use's manual (ELISA kits, eBioscience).

### Histological evaluation

Twenty-four hours after the last allergen challenge, lungs were harvested, fixed in 10% neutral-buffered formalin and embedded in paraffin. Sections (5 μm) of specimens were put onto 3-amino propyltriethoxy silane 3-Aminopropyltriethoxysilane-coated slides. The tissues were assessed for morphology and cellular infiltration using haematoxylin and eosin (H&E) staining. The degree of cellular infiltration was scored as described previously [6]. Inflammatory changes were graded by histopathological assessment using a semiquantitative scale of 0-5 for perivascular eosinophilia, bronchiolar eosinophilia, epithelial damage and oedema.

### Generation of DCs from spleen and culture

Spleen DCs were enriched as described previously [7]. Briefly, after the spleen was disrupted, the cells were centrifuged at 1300 rpm for 5 min, resuspended in RPMI 1640 medium supplemented with 10% heat-activated fetal calf serum, 2 mmol/l L-glutamine, 1 mmol/l pyruvate, 50 μmol/l mercaptoethanol, 100 U/ml penicillin, and 100 μg/ml streptomycin, and then incubated in plastic cell cultures plates for 2 h at 37°C in a 5% CO_2 _atmosphere. Culture plates were then washed thrice with RPMI 1640 medium and nonadherent cells were discarded. The residual adherent cells were maintained in the culture medium and incubated overnight at 37°C in a 5% CO2 atmosphere. After incubation, DCs with the adherence capacity in the first hours of culture become nonadherent and float in the medium. The DCs were collected and immediately used in the assays.

### Detection of CD_11c_^+^CD_80_^+^and CD_11c_^+^CD_86_^+^surface markers on spleen DCs

DCs were harvested from the spleen, incubated with FITC-labeled CD_11c _(eBioscience Inc.), PE-labeled anti-CD_80 _(B7-1) mAb (eBioscience Inc.), and PE-labeled anti-CD_86 _(B7-2) mAb (Southernbiotech) on ice for 30 min, and washed with PBS thrice. Ten thousand cells were collected from each sample and the data were analyzed with flow cytometer and CELLQUEST software (Coulter, Becton Dickinson, USA). For the DC marker staining, DCs were incubated with FITC-labeled rat anti-mouse IgG as isotype control on ice for 30 min and washed. The expression of the costimulatory molecules, i.e. CD_11c_^+^CD_80_^+^and CD_11c_^+^CD_86_^+^, surface markers on spleen DCs detected by FACS.

### Detection of peripheral CD_4_^+ ^and CD_8_^+ ^T cells

Blood from mice was transferred into 6 × 50 ml Falcon tubes using a 50 ml stripette and centrifuged at 1800 rpm for 5 min. The supernatant was aspirated and pooled into two of the tubes with PBS for a total volume of 75 ml. The 15 ml of Ficoll Paque was placed into three 50 ml tubes/group.

25 ml of the diluted filter material was transferred onto the Ficoll Paque so that they form two separate layers. After the centrifugation at 1800 rpm for 20 min, the leukocytes can now be collected at the interphase between the Ficoll Paque and the plasma. The CD_4_^+ ^and CD_8_^+ ^T cells were isolated from the peripheral blood were detected by flow cytometry. After autologous CD_4_^+ ^and CD_8_^+ ^T cells were stimulated with the targeted DCs, the proliferation and cytokine production were measured. Flow-cytometry assay was carried to detect the numbers of peripheral CD_4_^+ ^and CD_8_^+ ^T cells in mice 24 h after the last OVA challenge, using FITC-labeled CD_4_^+ ^and CD_8_^+ ^mAb, hemolytic agent( BD PharMingen) and IgG2a (Sero Tec).

### Detection of Foxp_3_^+^Tregs in spleen CD_4_^+ ^T cells

We harvested CD_4_^+ ^T cells of spleen from the various groups using antibody-coated paramagnetic MultiSort MicroBeads (MACS, Miltenyi Biotec, Bergisch Gladbach, Germany) according to the manufacturers protocol. After detaching, CD_4_^+ ^T cells were stained with CD_25 _MicroBeads (Miltenyi Biotec) and CD_4_^+^CD_25_^+^T cells were positively and negatively selected. Separation was controlled by FCM and Spleen CD_4_^+ ^T cells were labeled with Mouse Regulatory T cell Staining Kit to detect the expression of Foxp_3_^+ ^in spleen CD_4_^+ ^T cells (eBioscience Inc.).

### Statistical analysis

Data were expressed with means ± SD. Difference between groups was analyzed using software SPSS for windows (version 8.0) by unpaired two-tailed parametric Student's *t*-test or ANOVA test. *P*-values less than 0.05 were considered statistically significant.

## Results

**Figure 2:**
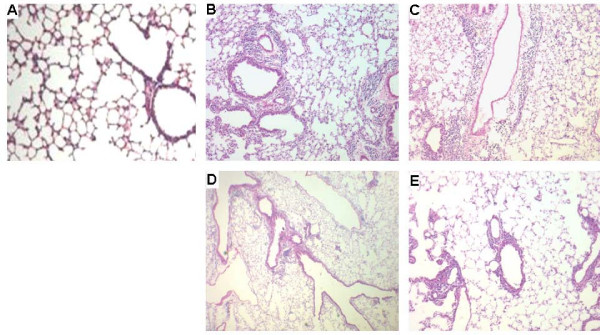
**Histological findings of peripheral airway tissues (H&E, ×100 origin) in PBS-vaccinated mice sensitized and challenged with PBS (A), PBS-vaccinated mice sensitized and challenged with ovalbumon (OVA) (B), pcDNA_3.1_-vaccinated mice sensitized and challenged with OVA (C), OVA- pcDNA_3.1_-vaccinated mice sensitized and challenged with OVA (D), and OVA-Fc-pcDNA_3.1_-vaccinated mice sensitized and challenged with OVA (E)**.

Histological analysis demonstrated that significant airway inflammation was observed in OVA-sensitized and challenged animals vaccinated with PBS or pcDNA_3.1 _plasmid, but less with OVA- pcDNA_3.1 _or OVA-Fc-pcDNA_3.1 _vaccination. The severity of leukocyte infiltration around the central bronchi, alveoli and blood vessels was scored and shown in Fig. 2. Leukocyte infiltration of the lungs was reduced by vaccination with OVA plasmid compared to vector alone. The thickness of small airway walls and the number of infiltrated eosinophils around the airway increased, eosinophils appeared within the lumen of the airway, and goblet cell hyperplasia and hypertrophy occurred in OVA-sensitized and challenged animals vaccinated with PBS (Fig. 2B) or pcDNA_3.1 _(C), as compared with those sensitized and challenged with PBS (A). OVA challenge led to a dense inflammatory infiltrate of lymphocytes, mononuclear and eosinophils as well as to epithelial shedding, which was partially prevented by immunization with pcDNA_3.1 _or OVA- pcDNA_3.1 _(Fig. 2D) and obviously by OVA-Fc-pcDNA_3.1 _(Fig. 2E).

**Figure 3:**
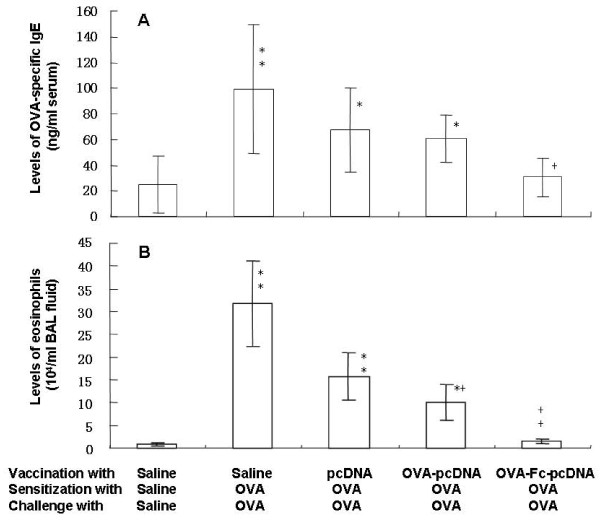
**Serum levels of OVA-specific IgE (A) and the number of eosinophils in bronchoalveolar lavage (BAL) fluid (B) in PBS-vaccinated mice sensitized and challenged with PBS, PBS-vaccinated mice sensitized and challenged with ovalbumon (OVA), pcDNA_3.1_-vaccinated mice sensitized and challenged with OVA, OVA- pcDNA_3.1_-vaccinated mice sensitized and challenged with OVA, and OVA-Fc-pcDNA_3.1_-vaccinated mice sensitized and challenged with OVA**. * and ** stand for the p values less than 0.05 and 0.01, respectively, as compared with PBS-vaccinated mice sensitized and challenged with PBS. + and ++ stand for the p values less than 0.05 and 0.01, respectively, as compared with OVA-Fc-pcDNA_3.1_-vaccinated mice sensitized and challenged with OVA.

**Figure 4:**
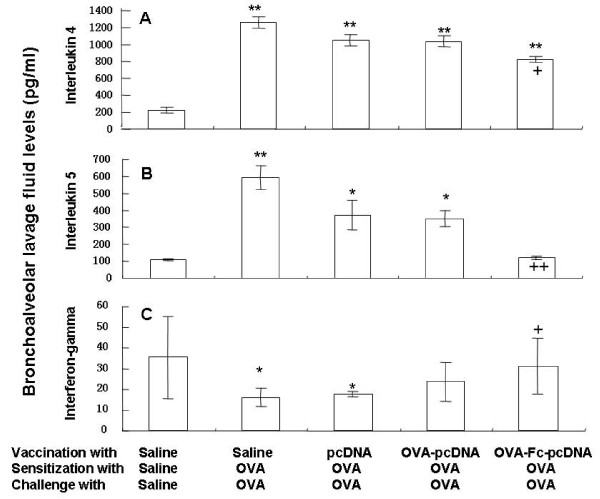
**Levels of interleukin-4 (A), interleukin-5 (B) and interferon gamma (C) in bronchoalveolar lavage (BAL) fluid (B) in PBS-vaccinated mice sensitized and challenged with PBS, PBS-vaccinated mice sensitized and challenged with ovalbumon (OVA), pcDNA_3.1_-vaccinated mice sensitized and challenged with OVA, OVA- pcDNA_3.1_-vaccinated mice sensitized and challenged with OVA, and OVA-Fc-pcDNA_3.1_-vaccinated mice sensitized and challenged with OVA**. * and ** stand for the p values less than 0.05 and 0.01, respectively, as compared with PBS-vaccinated mice sensitized and challenged with PBS. + and ++ stand for the p values less than 0.05 and 0.01, respectively, as compared with OVA-Fc-pcDNA_3.1_-vaccinated mice sensitized and challenged with OVA.

As shown in Fig. 3A, serum levels of OVA-specific IgE were significantly higher in OVA-sensitized and challenged mice vaccinated with PBS, as compared with these sensitized, challenged and vaccinated with PBS (p < 0.01, Fig. 3A). OVA-sensitized and challenged animals vaccinated with OVA-Fc-pcDNA_3.1 _had significantly lower levels of OVA-specific IgE, as compared with those vaccinated with PBS or pcDNA_3.1 _(p < 0.05). Animal vaccinated with pcDNA _3.1 _plasmid had similar levels of OVA-specific IgE with those vaccinated with OVA-pcDNA_3.1_. The number of eosinophils in BAL fluid of OVA mice vaccinated with PBS or pcDNA _3.1 _was significantly higher than those without OVA (p < 0.01, Fig. 3B). Immunization with OVA-Fc-pcDNA_3.1 _significantly prevented OVA-induced eosinophilia in BAL fluid, as compared with that with PBS, pcDNA_3.1 _or OVA-pcDNA_3.1 _(p < 0.01 or 0.05, respectively). Vaccination with OVA-pcDNA_3.1 _partially prevented from OVA-increased number of eosinophils (p < 0.05, vs PBS or pcDNA_3.1_, respectively). OVA induced a significant elevation of IL-4 BAL fluid in all animals, as compared with animals without OVA (p < 0.01 or 0.05, respectively, Fig. 4A), while OVA-Fc-pcDNA_3.1 _showed partially preventive effects (p < 0.05, vs OVA animals vaccinated with PBS). The BAL levels of IL-5 in OVA animals vaccinated with PBS, pcDNA_3.1 _or Fc-pcDNA_3.1 _were significantly higher than those without OVA, which was significantly prevented by OVA-Fc-pcDNA_3.1_, as compared with pcDNA_3.1 _or Fc-pc (p < 0.01, respectively, Fig. 4B). Immunization with OVA-Fc-pcDNA_3.1 _also significantly prevented OVA-reduced level of INF-γ in BAL fluid, as immunization with PBS or pcDNA_3.1 _(p < 0.05, Fig. 4C).

**Figure 5:**
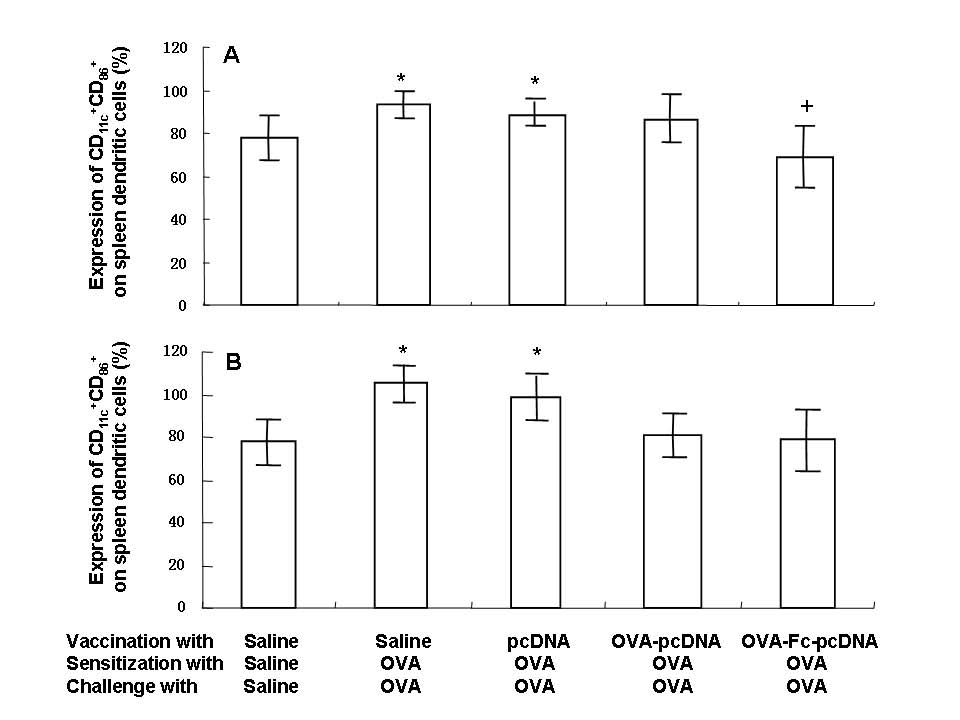
**Expression of CD_11c_^+^CD_80_^+ ^(A) and CD_11c_^+^CD_86_^+ ^(B) on spleen dendritic cells harvested from PBS-vaccinated mice sensitized and challenged with PBS, PBS-vaccinated mice sensitized and challenged with ovalbumon (OVA), pcDNA_3.1_-vaccinated mice sensitized and challenged with OVA, OVA- pcDNA_3.1_-vaccinated mice sensitized and challenged with OVA, and OVA-Fc-pcDNA_3.1_-vaccinated mice sensitized and challenged with OVA**. * stands for the p values less than 0.05, as compared with PBS-vaccinated mice sensitized and challenged with PBS. + stands for the p values less than 0.05, as compared with OVA-Fc-pcDNA_3.1_-vaccinated mice sensitized and challenged with OVA.

We isolated spleen-derived DCs from all animals, identified the characteristics of these DCs expressed CD_11c _molecule, detected OVA-increased expression of CD_80_^+ ^and CD_86_^+ ^on DCs obtained from the spleen, and then evaluated the preventive effects of OVA-Fc-pcDNA_3.1 _on the expression of CD_80_^+ ^and CD_86_^+ ^on DCs obtained from the spleen of OVA-sensitized and challenged mice. OVA induced a significant expression of CD_11c_^+^CD_80_^+ ^(Fig. 5A) and CD_11c_^+^CD_86_^+ ^(Fig. 5B) on spleen DCs harvested from mice vaccinated with PBS or pcDNA_3.1_, as compared with those without OVA (p < 0.05), which was significantly prevented by the vaccination with OVA-Fc-pcDNA_3.1_.

**Figure 6:**
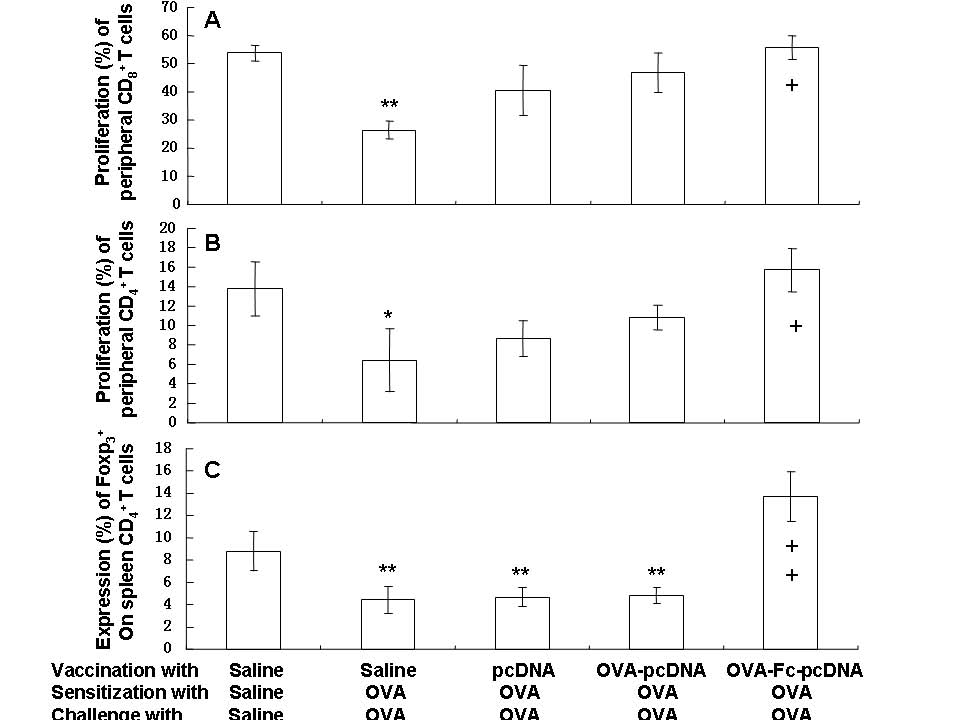
**Proliferation of peripheral CD_4_^+ ^(A) and CD_8_^+ ^T cells (B) and the expression of Foxp_3_^+ ^on spleen CD_4_^+ ^T cells (C) harvested from PBS-vaccinated mice sensitized and challenged with PBS, PBS-vaccinated mice sensitized and challenged with ovalbumon (OVA), pcDNA_3.1_-vaccinated mice sensitized and challenged with OVA, OVA- pcDNA_3.1_-vaccinated mice sensitized and challenged with OVA, and OVA-Fc-pcDNA_3.1_-vaccinated mice sensitized and challenged with OVA**. * and ** stand for the p values less than 0.05 and 0.01, respectively, as compared with PBS-vaccinated mice sensitized and challenged with PBS. + and ++ stand for the p values less than 0.05 and 0.01, respectively, as compared with OVA-Fc-pcDNA_3.1_-vaccinated mice sensitized and challenged with OVA.

Our pilot study showed that targeted DCs stimulated the proliferation of peripheral CD_4_^+ ^T and CD_8_^+ ^T cells in a concentration-dependent pattern. The proliferation of both peripheral CD_8_^+ ^(Fig. 6A) and CD_4_^+ ^T cells (Fig. 6B) in OVA mice vaccinated with PBS or pcDNA_3.1 _was significantly lower than those without OVA (p < 0.01 or 0.05, respectively). Vaccination with OVA-Fc-pcDNA_3.1 _significantly prevented OVA-suppressed cell proliferation (p < 0.05). The expression of Foxp_3_^+ ^on spleen CD_4_^+ ^T cells were significantly suppressed by OVA mice vaccinated with PBS, pcDNA_3.1 _and Fc-pcDNA_3.1 _(p < 0.01, respectively, Fig. 6C), but not with OVA-Fc-pcDNA_3.1_.

## Discussion

The results from the present study demonstrated that allergen-DNA-targeted DCs were highly effective in preventing allergen-induced airway inflammation in a murine allergic model. OVA-sensitization and challenge could induced the development of airway inflammation and Th2 responses and suppressed the proliferation of peripheral CD_4_^+^and CD_8_^+ ^T cells and the expression of Foxp_3_^+ ^Treg in spleen, which could be prevented by the vaccination with OVA-Fc-pcDNA_3.1. _The potential mechanisms of OVA-Fc-pcDNA_3.1 _effects may be that Fc enhancing DC absorption is more powerful in stimulating the production of T_H_1 cytokine synthesis in naïve and memory T cells.

As an alternative to the administration of allergens or allergen derivatives, vaccination with allergen-encoding DNA has been proposed as a strategy for SIT [8-10]. Dendritic cells are uniquely situated in the immune cascade to initiate and modulate airway immune responses [11-13]. In addition, immature DCs express a number of specific chemokine receptors, FcR, and Toll-like receptors, through which the DCs are developing [14-16]. DCs can be activated or inhibited through FcR by antibodies or immune complex formed by antibodies depending on the variations of FcR engaged. Activating and inhibitory IgG Fc receptors on DCs mediate opposing functions [17,18] DNA vaccination may directly target DCs involved in allergen-specific T_H_1-cell responses, showing preventive effects when delivered in mice [19-23]. The vaccination with OVA-pcDNA_3.1 _or OVA-Fc-pcDNA_3.1 _showed preventive effects on OVA-induced hyper-production of IL-4 and IL-5 and hypo-production of INF-γ, and eosinophil infiltration, while the effects of OVA-Fc-pcDNA_3.1 _were more significant. Our study suggested that DNA vaccine encoding both mouse Fc and OVA was more effective than DNA vaccine encoding only OVA in suppressing airway inflammation.

We found that local exposure to OVA resulted in an increased number of spleen DCs expressing CD_11c_^+^CD_80 _and CD_11c_^+^CD_86 _molecules, similar to the previous findings [24], suggesting that CD_80 _and CD_86 _molecules act as the regulators of immune responses. The CD_80 _and CD_80_, as the most important costimulatory molecules, could play the important role in the allergic immune responses, indicating that the effective cross-presentation and DC maturation should be considered in the development of efficacious targeting strategies. Our results also showed that OVA-induced Tregs decrease could also be prevented by the allergen-DNA-targeted-DCs, suggesting that the allergen-DNA-targeted-DC may be useful in SIT and the restoration of Tregs played a key role in successful SIT.

Costimulatory molecules as potential targets contribute to the therapeutic intervention in allergic airway disease, which could be treated with DCs activated by cross-linking B7-DC [25-28]. Spleen DCs function could be modulated following SIT. Immune complexes of IgG and antigen can be internalized via FcRs on DC, resulting in DC maturation and priming of antigen-specific CD_8_^+^T cells in vivo[29,30]. However, DCs express both activating and inhibitory FcRs, and the balance between activating and inhibitory signaling will determine whether uptake of immune complexes results in naive T cell activation and protective immunity [31]. The OVA-Fc-pcDNA_3.1 _influenced those surface marker expressions on targeted DCs, probably down-regulating general capability of DCs to present antigen [32-34]. Spleen DCs have a partially mature phenotype and express a range of co-stimulatory molecules that are intermediate between immature and mature DCs, resulting in tolerogenic interaction with T cells in SIT. We found that the combination of DNA vaccination with Fc and OVA had better effects onOVA-induced alterations in the spleen. It is evidenced by the previous findings that DNA vaccination suppressed both Th1 and Th2 responses (IFN-γ and IL-4 production from spleen cells) [34-36]. It would be more interesting to explore the potential mechanisms of CD_11c_^+^CD_80 _and CD_11c_^+^CD_86 _induction and regulation between DCs and Tregs in the further studies.

A great number of studies have shown that the targeting of antigens to DC surface receptors elicits effective immune responses [37-42], although it is still questionable that the certain surface receptors can make more suitable targets than others. Immunization with OVA-Fc-pcDNA_3.1 _could prevent OVA-induced over-formation of allergen-specific IgE, local hyper-production of IL-4 and IL-5 and hypo-production of INF-γ, over-expression of costimulatory molecules CD_11c_^+^CD_80_^+^and CD_11c_^+^CD_86_^+^in spleen, and finally airway inflammation. Thus, DNA vaccine encoding OVA directly to DCs may a new alternative of therapies for patients with allergic asthma. These findings suggest that spleen DCs and Foxp_3_^+^Tregs prevents the generation and activation of Th2 effector cells as a novel pathway of regulation of type 2 immunity in asthma.

## Competing interests

The authors declare that they have no competing interests.

## Authors' contributions

YL performed all analyses and wrote the initial draft of the paper. XM obtained funding for the project, conceived the question, and directed writing and analysis.  YL, GS, XM, CX, and XD participated in funding, data collection, data analysis and interpretation, and editing. J and DP participated in the reversed manuscript. All authors have read and approved the final manuscript.
